# In vitro antibacterial activity of *Loxostylis alata* extracts and isolated compounds against *Salmonella* species

**DOI:** 10.1186/s12906-021-03292-4

**Published:** 2021-04-13

**Authors:** Dorcas A. Gado, Muna Ali Abdalla, Aroke S. Ahmed, Balungile Madikizela, Sanah M. Nkadimeng, Marthie M. Ehlers, Lyndy J. McGaw

**Affiliations:** 1 Phytomedicine Programme, Department of Paraclinical Sciences, University of Pretoria, Private Bag X04, Onderstepoort 0110, Pretoria, South Africa; 2 Regional Laboratory for Animal Influenzas and other Transboundary Animal Diseases, National Veterinary Research Institute, PMB 01, Vom, Plateau State Nigeria; 3 Department of Food Science and Technology, Faculty of Agriculture, University of Khartoum, 13314 Khartoum North, Sudan; 4 Department of Medical Microbiology, Faculty of Health Sciences, University of Pretoria, PO Box X323, Arcadia 0007, Pretoria, South Africa; 5 National Health Laboratory Service, Tshwane Academic Division, Pretoria, South Africa

**Keywords:** Antimicrobial, *Salmonella*, Cytotoxicity, *Loxostylis alata*, Biflavonoid

## Abstract

**Background:**

Owing to antibiotic resistance, alternative antimicrobials from medicinal plants are receiving attention as leads for anti-infective agents. This study aimed to investigate selected tree species and their constituents for activity against bacterial foodborne pathogens, particularly *Salmonella* serovars.

**Methods:**

Antibacterial activity of ten plant species was determined by serial microdilution against bacteria implicated in causing gastrointestinal ailments. Active compounds were isolated from *Loxostylis alata* using bioassay-guided fractionation. Antioxidant activity was determined using free-radical scavenging assays. Cytotoxicity and genotoxicity of the extracts was ascertained on Vero cells, and using the Ames assay respectively.

**Results:**

Extracts had low to moderate MIC values from 0.04 to 2.5 mg/mL. *Protorhus longifolia* and *Loxostylis alata* were most active and *L. alata* had the highest selectivity index value (2.51) against *Salmonella* Typhimurium, as well as high antioxidant activity. Cytotoxicity values ranged from 0.02 to 0.47 mg/mL, while tested extracts were not genotoxic. Bioactive compounds isolated from *L. alata* included delicaflavone and a polymethoxyflavone.

**Conclusions:**

The *Loxostylis alata* leaf extract had strong activity against *Salmonella* serovars but isolated compounds were less active, indicating likely synergistic effects. Extracts of *L. alata* are promising candidates for development of antimicrobial preparations or food additives against microbial contamination.

## Background

Microbial safety of food is a major concern to consumers, regulatory agencies and food industries throughout the world [1]. About 600 million people globally suffer from ill health due to consumption of contaminated food, and about 420,000 deaths are recorded annually (WHO, 2018a). Foodborne diseases of bacterial aetiology are commonly caused by enterohaemorrhagic *Escherichia coli* as well as *Campylobacter* and *Salmonella* species [2]. *Salmonella* species are considered second only to *Campylobacter* as a major cause of gastrointestinal infections in companion animals, livestock and humans, particularly in developing countries [3]. *Salmonella* contamination and resultant infection has a high economic burden in food production, due to a very broad host range and significant morbidity and mortality in the human population [4, 5]. Presently, over 2500 *Salmonella* serovars have been characterized but only a few (approximately 150) cause salmonellosis in humans and domestic animals, and all motile serovars are reported as zoonotic [6]. *Salmonella enterica* subsp. *enterica* serotype Typhimurium (*Salmonella* Typhimurium) and *Salmonella* Enteritidis are the most common causes of human infection [7]. In 2015 alone, invasive non-typhoidal *Salmonella* (iNTS) disease was estimated to cause 680,000 deaths per year worldwide, more than half of which were in Africa [8]. Therefore, *Salmonella* control will continue to be a major task at all stages of the food chain from production through to processing, distribution and consumption, to decrease the incidence of food contamination and to ensure food safety [9].

Gastroenteritis is mostly treated with a wide range of antibiotics, usually administered at reduced or prophylactic levels to enhance performance and prevent infection in animals [10–12]. The indiscriminate use of antibiotics as prophylaxis and growth enhancers in animal production is reported to have contributed to the current global challenge of emergence of drug-resistant and multidrug-resistant strains of bacteria, threatening global health and food security [13, 14]. Humans acquire resistant serovars from food or animals, and subsequent transmission occurs from human to human [15]. Resistance to antibiotics complicates treatment through higher treatment costs and increased mortality [16], especially in immunocompromised individuals. These undesired consequences of antibiotic resistance, coupled with the absence of effective oral vaccines protecting against salmonellosis, have resulted in consumers demanding safe, high-quality foods and hence researchers have been searching for alternative methods to control enteric infections [17, 18]. It has therefore become imperative to search for novel antimicrobials effective against resistant pathogens, or alternatively, immune modulators or other biologics to combat pathogens [19–23].

As bacteria continue to evolve, there is growing global attention towards exploring the natural world for secondary metabolites as potential sources for drug development [22]. Several drugs have been developed over time from natural products [24–26]. Plant extracts contain mixtures of secondary metabolites such as alkaloids, coumarins, flavonoids, glycosides, phenols, quinones, saponins, steroids, tannins and terpenoids [27, 28]. Each of these metabolites may possess individual bioactivity or may act together in synergy to disrupt the growth or pathogenesis of disease-causing organisms [29]. Polyphenolic compounds are major constituents of medicinal plants reported to act as free radical scavengers and antioxidants [30, 31].

In this study, selected South African plant species known to have good efficacy against Gram-negative *Escherichia coli* [32] were screened for antibacterial activity against strains implicated in foodborne diseases as a lead to identifying extract(s) or compound(s) active against *Salmonella* species. Plant extracts with good antimicrobial activity were evaluated for genotoxicity and cytotoxicity, and antioxidant activity, which is an additional beneficial property of medicinal plants. The use of active plant components as natural additives has gained increasing interest in the food and health industries, with recent studies promoting the possibility of using these antioxidant and antimicrobial constituents as food preservatives, or functional food ingredients [33, 34]. Owing to promising preliminary activity (good antimicrobial activity, relative safety and good antioxidant activity), extracts of *L. alata* were further investigated for their antibacterial activity against commercially available *Salmonella enterica* serovar Typhimurium (ATCC 14028), and *S*. Enteritidis (ATCC 13076) as well as field isolates of *S.* Typhimurium, *S.* Dublin and *S.* Braenderup, with the aim of isolating compounds active against *Salmonella* serovars.

## Methods

### Plant collection and sample preparation

Leaves of *Blighia unijugata*, *Carissa macrocarpa*, *Combretum bracteosum*, *Kirkia wilmsii*, *Loxostylis alata*, *Noltea africana* and *Protorhus longifolia* were collected at the Manie van der Schijff Botanical Garden of the University of Pretoria, South Africa. *Brachychiton acerifolium*, *Brachychiton bidwillii* and *Searsia leptodictya* were collected at the Onderstepoort campus, Faculty of Veterinary Science, University of Pretoria, South Africa. All plants used in this study were collected in summer months of 2016. Voucher specimens of the plants were identified and deposited at the H.G.W.J. Schweickerdt Herbarium of the Department of Plant and Soil Sciences, University of Pretoria, South Africa. Leaves of plants collected were thoroughly cleaned and air- dried in a drying room away from direct sunlight, at the Department of Paraclinical Sciences, University of Pretoria. Thereafter, dried plants were ground to a fine powder in a Macsalab mill [(Model 200 LAB), Eriez, South Africa] and stored in closed containers in the dark until used.

### Preparation of plant extracts for in vitro biological assays

Powdered leaf material (10 g) of each plant was extracted separately by adding 100 mL each of acetone, methanol, ethanol (Minema, South Africa), cold and hot (boiling temperature) distilled water to obtain the respective crude extracts. The plant material in the extraction solvents was left to stand for 24 h and filtered through Whatman No. 1 filter paper (Merck, United States). The resultant extracts were transferred into pre-weighed labelled glass jars and the extraction procedure described above was repeated twice on the same plant material for exhaustive extraction. Pooled extracts were placed under a stream of air to dry completely and stored in the dark at 4 °C until needed for experiments.

**Fig. 1:**
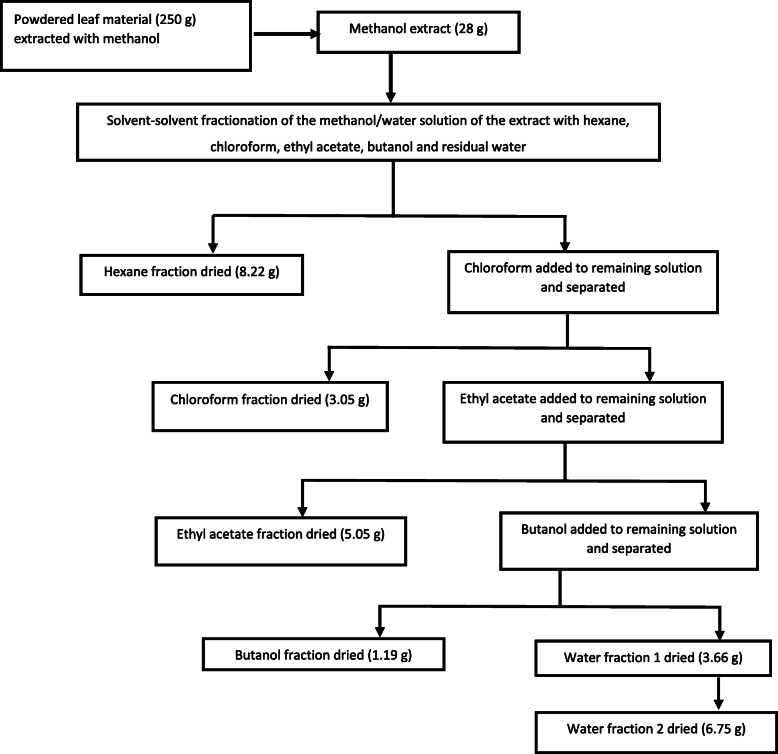
Procedure for solvent–solvent fractionation of methanol leaf extract of *Loxostylis alata*

Bulk extraction was carried out for compound isolation. Ground leaf material of *Loxostylis alata* (250 g) was extracted with 100% methanol (ratio 1:10) and extraction was repeated twice on the same plant material. The solvent of the combined extracts was removed in vacuo after filtration*.* The dried methanol extract (28 g) was subjected to solvent-solvent fractionation using solvents of different polarity: hexane, chloroform, ethyl acetate, butanol (Minema, South Africa) and water (Fig. 1).

### Antimicrobial screening

#### Microbial strains

Five of the bacterial species used in this study were obtained from the American Type Culture collection (ATCC) and consisted of Gram-positive strains: *Bacillus cereus* (ATCC 21366), *Enterococcus faecalis* (ATCC 29212) and *Staphylococcus aureus* (ATCC 29213), and Gram-negative strains: *Pseudomonas aeruginosa* (ATCC 27853) and *Salmonella* Typhimurium (ATCC 700720). The other bacteria were Gram-negative clinical isolates from eggs (Jambalang et al., 2017) and included *Enterobacter cloacae, Escherichia coli, Klebsiella pneumoniae, Proteus mirabilis* and *Stenotrophomonas maltophilia* obtained from the Phytomedicine Repository at the University of Pretoria.

For further evaluation of *L. alata*, two strains from the American Type Culture Collection, *Salmonella* Typhimurium (ATCC 14028) and *S*. Enteritidis (ATCC 13076), and local isolates of *S.* Dublin, *S.* Braenderup and *S.* Typhimurium provided by the Phytomedicine Programme, University of Pretoria [35] were included.

#### Culturing microbial strains

All bacterial strains were plated from cultures which were stored at − 80 °C on ceramic beads in cryoprotective media (Pro-Lab diagnostics Microbank^@^ 20) onto Müller-Hinton (MH) agar (Merck, South Africa) except for the *Salmonella* species which were plated unto xylose lysine deoxycholate (XLD-Merck, South Africa) and grown for 18 -24 h (IncoTherm; Labotec) at 37 °C. Cultures were subsequently sub-cultured and maintained on MH agar plates at 4 °C until needed for further antimicrobial testing.

#### In vitro *antibacterial assay*

The minimum inhibitory concentration (MIC) values of the plant extracts against different bacterial species were determined using a serial microdilution assay [36]. All bacterial isolates screened were prepared by inoculating a single colony from an agar plate into 10 mL of sterilized MH broth (Merck, South Africa) and incubated at 37 °C in an MRC orbital shaker (150 rpm) incubator (United Scientific, South Africa) for 18 to 20 h prior to the experiment. Following incubation, each bacterial strain was diluted in MH broth (Merck, South Africa) and the absorbance was measured at a wavelength of 560 nm using a spectrophotometer (Epoch microplate reader: BioTek, United States). Absorbance was adjusted to match that of a McFarland standard No 1 (correlating to approximately 3 × 10^8^ colony forming units (cfu)/mL). Sterile distilled water (100 μL) was added to each well of a 96-well microtitre plate (Lasec, South Africa). Plant extracts were re-constituted to 10 mg/mL in sterile distilled water for the water extracts and 70% acetone for the organic solvent extracts. Aliquots (100 μL) of each extract were added to the first well (row A) of the 96-well microtitre plates, and serially diluted two-fold down the wells to row H and the last 100 μL discarded. Gentamicin (Virbac, South Africa) and tetracycline (Sigma-Aldrich, United States) at an initial concentration of 2 mg/mL were used as positive controls, while acetone, sterile distilled water and bacterial cultures were used as negative and untreated controls. Subsequently, 100 μL of each diluted bacterial culture were added to all wells of the microtitre plates. The microtitre plates were incubated at 37 °C for 18 to 24 h (IncoTherm, Labotec). After incubation, 40 μL of 0.2 mg/mL p-iodonitrotetrazolium (INT) (Sigma-Aldrich, United States) were added to each well to determine the MIC and the microtitre plates were re-incubated at 37 °C for 1 h. Bacterial cultures react with INT and give a red or purple colouration within 10 to 60 min [36]. The last clear well was taken as the MIC, i.e. where bacterial growth was inhibited, the solution in the well remained clear or had a decreased colour change after incubation with INT. The experiment was repeated twice with three replicates in each assay. Besides determining the MIC for each plant extract, the total activity was calculated as “the total mass in mg extracted from 1 g of plant material divided by the MIC value (mg/mL)”. The total activity expressed in mL/g indicates “the volume to which the extract derived from 1 g of plant material can be diluted and still inhibit the growth of the microorganism” [37, 38].

### Antioxidant assays

#### The ABTS [2, 2–azino-bis- (3-ethylbenzothiazoline-6-sulfonic acid)] assay

The ABTS antioxidant assay was conducting following the described method [39], with Trolox, a synthetic water soluble vitamin E analogue, and ascorbic acid (Adminide, South Africa) serving as positive controls. The negative control was 100% methanol (Minema, South Africa), and extracts without ABTS served as blanks [38]. The percentage of ABTS^+^ inhibition was calculated using the following formula:

$$ \mathrm{Scavenging}\ \mathrm{capacity}\ \left(\%\right)=100\hbox{-} \left[\frac{\mathrm{Abs}\left(\mathrm{sample}\right)\hbox{-} \mathrm{Abs}\left(\mathrm{sample}\ \mathrm{blank}\right)}{\mathrm{Abs}\left(\mathrm{control}\right)\hbox{-} \mathrm{Abs}\left(\mathrm{control}\ \mathrm{blank}\right)}\times 100\right] $$

IC_50_ values were calculated from the graph plotted as percentage inhibition against the concentration [38].

#### The DPPH (2, 2′-diphenyl-1-picrylhydrazyl) assay

The DPPH assay was conducted according to the method described by [40] with ascorbic acid and Trolox as positive controls. Methanol (100%) was used as a negative control and extracts without DPPH (2, 2′-diphenyl-1-picrylhydrazyl) served as blanks. Results were expressed as the concentration of extract that reduced the DPPH colour by 50% (IC_50_), which was determined as for the ABTS assay [38].

### Toxicological assays

#### In vitro *cytotoxicity*

The MTT (3-(4, 5-dimethylthiazolyl-2)-2.5-diphenyltetrazolium bromide) reduction assay was used to measure cell proliferation and cytotoxicity of plant extracts against African green monkey kidney (Vero) cells (ATCC® CCL-81™, Sigma-Aldrich, United States). The viable cell growth after incubation with test plant extracts was determined using the tetrazolium-based colorimetric assay (MTT assay) [41] with some modification [42]. Vero cells were grown in sterile Minimal Essential Medium (MEM) (Sigma-Aldrich, United States) supplemented with 0.1% gentamicin (Virbac, South Africa) and 5% foetal calf serum (FCS) (Highveld Biological, South Africa) in a 75 cm^2^ flask (Whitehead Scientific, South Africa), and incubated at 37 °C in 5% CO_2_ (Hera Cell 150, ThermoScientific Germany). When cells had grown to confluency, they were harvested using trypsin/ethylenediamine tetraacetic acid (EDTA) solution (Invitrogen, Cergy-Pontoise, France), centrifuged (Universal 320R, Labotech South Africa) for 5 min at 200×g, counted using a Neubauer haemocytometer, and re-suspended in MEM. The cells were seeded in a 96-well cell culture grade microtitre plate (Whitehead Scientific, South Africa) at a final concentration of 10,000 cells per well. The plates were incubated at 37 °C in 5% CO_2_ overnight to allow proper attachment of cells and to enable cells to reach exponential growth phase.

A stock concentration of 100 mg/mL of the plant extract was prepared, from which different concentrations were prepared in serum-free MEM ranging from 0.0075 to 1 mg/mL, and the cells were treated in triplicate. Doxorubicin hydrochloride (Pfizer Laboratories, South Africa) was used as a positive control, whereas untreated cells and 1% dimethyl sulphoxide (DMSO) were used as negative controls. The plates were incubated at 37 °C in 5% CO_2_ environment (Hera Cell 150, ThermoScientific Germany) for 48 h. After the 48 h incubation period, the medium was aspirated from the wells, and the cells were washed with 200 μL of phosphate buffered saline (PBS) pH 7.4 (Whitehead Scientific, South Africa). Fresh MEM (100 μL) and 30 μL of MTT (5 mg/mL stock solution: Sigma-Aldrich, South Africa) dissolved in PBS were added to all the wells and the plates were incubated at 37 °C in 5% CO_2_ for 4 h. Following incubation, the media with MTT was removed, 50 μL of DMSO (BDH, South Africa) were added to each well, and the plates were shaken gently (QG-9001 microporous Quickshaker, Hinotek; China) until the crystals were dissolved. The amount of MTT reduction was measured using a spectrophotometer (Biotek Synergy, USA) at a wavelength of 570 nm (reference wavelength of 630 nm). The wells in column 1, containing medium (MEM) and MTT but no cells were used as blanks. The results were interpreted as percentage of the control wells, and lethal concentration (LC_50_) values calculated as the concentration of plant samples resulting in a 50% reduction of absorbance after 48 h incubation (that is 50% of the cells were killed) compared to untreated cells. The selectivity index values for each extract were calculated from the MIC values of the extracts against tested bacterial strains and LC_50_ values, using the following formula: SI = LC_50_**/**MIC.

#### In vitro *genotoxicity assay*

In this study, extracts that showed MIC values less than 1 mg/mL against at least three bacterial strains were tested for genotoxicity. The *Salmonella* microsome assay according to Maron and Ames (1983) and modified by Mortelmans and Zeiger (2000) was used to determine the genotoxicity of selected extracts. Two *Salmonella typhimurium* tester strains, TA98 and TA100 which indicate frame-shift and base pair mutations respectively, were used for the assay which was done without metabolic activation. Briefly, crude plant extract was dissolved in 10% DMSO (BDH, South Africa) and diluted to yield final concentrations of 5, 0.5 and 0.05 mg/mL. Approximately 1 × 10^8^ CFU stock bacterial cultures (100 μL) incubated in nutrient broth No. 2 (Sigma-Aldrich, South Africa) on a MRC orbital shaker (150 rpm) incubator (United Scientific, South Africa) at 37 °C for 16 h, were added to test samples (100 μL) followed by 500 μL of pH 7.4 PBS (Whitehead Scientific, South Africa) and 2 mL of top agar containing biotin/histidine (0.5 mM). A positive control, 4-nitroquinoline-1-oxide [(4-NQO) (Sigma-Aldrich, South Africa)] at a concentration of 2 μg/mL was used, and two negative controls, sterile distilled water and 10% DMSO (BDH, South Africa), were used. The mixture was vortexed (VM-300 vortex mixer, Gemmy; Taiwan), and poured on minimal agar plates (Sigma-Aldrich, South Africa) and incubated (IncoTherm; Labotec) for 48 h at 37 °C. Each sample was tested in triplicate for each concentration and the experiment was repeated 3 times. After 48 h of incubation (IncoTherm; Labotec) at 37 °C, revertant (mutant) colonies were counted. An extract is considered mutagenic when the mean number of revertants is equal to or more than double that found in the negative control [43, 44].

### Statistical analysis

Data are presented as mean ± standard error of mean. Differences in the means were subjected to post-hoc analysis using the Tukey’s test. The IBM SPSS software package version 25.0.0 was used for the statistical tests. Differences were considered significant at *p* < 0.05.

### Isolation of compounds

The dried methanol extract (28 g) was dissolved in a mixture of hexane and water and fractionated by solvent-solvent extraction (Fig. 1) to yield hexane (8.22 g), chloroform (3.05 g), ethyl acetate (5.05 g), butanol (1.19 g), water 1 (3.66 g) and water 2 (6.75 g) fractions after drying. The organic solvent fractions were evaporated to dryness under reduced pressure at low temperature (40 to 50 °C) in a rotary evaporator (Buchi Rotavapor R-200, Switzerland). The water fraction was dried by vaporisation in an oven at 60 °C.

The ethyl acetate fraction had the best antimicrobial activity against the *Salmonella* serovars used in this study and was therefore chosen for further isolation of active compounds. Column chromatography (CC) with silica gel 60 (Sigma-Aldrich, South Africa) was carried out to fractionate the ethyl acetate fraction (5.05 g) using a combination of chloroform: methanol: formic acid (90:10:2) (Minema, South Africa). Fractions with similar appearance and Rf values on TLC plates (Merck, United States) when viewed on a universal UV lamp (CAMAG TL-900/U, Switzerland), were pooled to obtain seven fractions (FI to FVII). Silica gel CC was further used to run the FII fraction using the same solvent combination but this time at a ratio of 95:5:2 and this yielded two pure whitish compounds (samples 1 and 2). The FI, FIII to FVII fractions were further combined and a chloroform: methanol step gradient Silica gel CC ran: starting with 100% chloroform and then reduced to 0% chloroform by the addition of methanol in successive steps, i.e. CHCl_3_: MeOH (100:0, 99:1, 97:3, 95:5, 93:7, 93:10, 87:13 and 85:15). Fractions with a similar appearance on TLC plates were pooled to obtain eight fractions (F1–8). The F7 fraction was a pure whitish yellow compound (sample 3). The F6 fraction was only partially soluble in methanol and was filtered using Whatman No.1 filter paper. The filtrate (methanol-soluble) was yellowish in solution while the residue/precipitate (acetone soluble) was dirty white in colour (sample 4). The filtrate was centrifuged (EBA 20, Labotec; South Africa) for 5 min at 3000 x g to obtain a clear supernatant and sediment. The supernatant was further fractionated by Sephadex LH-20 (Sigma-Aldrich, South Africa) column chromatography eluted isocratically with methanol to give a yellowish powder (sample 5).

### Structure identification of the isolated compounds

Compound detection was performed using a Waters® Synapt G2 high definition mass spectrometry (HDMS) system (Waters Inc., Milford, Massachusetts, USA). Samples were analysed using flow injection analysis (FIA). The system comprises of a Waters Acquity Ultra Performance Liquid Chromatography (UPLC®) system hyphenated to a quadrupole-time-of-flight (QTOF) instrument. The system was operated with MassLynxTM (version 4.1) software (Waters Inc., Milford, Massachusetts, USA) for data acquisition and processing. Nuclear magnetic resonance (NMR) (1D and 2D) spectroscopy was also carried out. Proton nuclear magnetic resonance (^1^H NMR) and two-dimensional nuclear magnetic resonance (2D NMR) data were acquired on a 400 MHz NMR spectrometer (Bruker Avance III 400 MHz). Structures of isolated compounds were confirmed by comparison of their NMR data with those published previously.

Samples not detected as pure compounds by NMR were subjected to gas chromatography coupled to a mass spectrometer (GC-MS) using a LECO Pegasus 4D GC-TOFMS (LECO Africa (Pty) Ltd., Kempton Park, South Africa). The GC column was a Rxi-5SilMS 30 m × 0.25 mm ID × 0.2 μm film thickness (Restek, Bellefonte, PA, USA). The injector port was maintained at 250 °C; the oven temperature was programmed at 40 °C (hold for 3 min) to 300 °C (hold for 5 min). The carrier gas was helium (helium UHP, Afrox, Gauteng, South Africa) and the velocity of the gas was at 1 mL/min in the constant flow mode. The MS transfer line was set to 280 °C, and the ion source temperature was 230 °C. The electron energy was 70 eV in the electron impact ionization mode, the mass acquisition range was from 40 to 550 Da, the detector voltage was held at 1750 V. Data acquisition at a rate of 10 spectra/s was done.

## Results

### Antibacterial activity

The botanical names of the plants, voucher reference numbers, and traditional uses and parts used are presented in Table 1. The results of the antibacterial assay of the fifty extracts from ten plant species against five ATCC bacterial strains and five clinical isolates are presented as MIC values in Table 2. MIC values of the extracts ranged from 0.04 to 2.5 mg/mL against all strains tested. The best MIC value of 0.04 mg/mL was observed with several extracts and others had good MIC values of 0.08 mg/mL. Overall for the crude extracts, the lowest sensitivity was obtained for *S*. Enteritidis (ATCC 13076) with a mean MIC value of 0.31 mg/mL.

**Table 1:** Botanical name, family and voucher specimen number of each plant species used in this study

Family	Plant species	Common name	Voucher specimen number
Anacardiaceae	*Loxostylis alata* A. Spreng. ex Rchb	Tarwood	PRU 124357
Anacardiaceae	*Protorhus longifolia* Bernh. (Engl.)	Red Beech	PRU 122537
Anacardiaceae	*Searsia leptodictya* (Diels) T.S.Yi, A.J.Mill. & J.Wen	Mountain karee	PRU 122531
Apocynaceae	*Carissa macrocarpa* (Eckl.) A.DC.	Big Num Num	PRU 122534
Combretaceae	*Combretum bracteosum* (Hochst.) Engl. & Diels	Hiccup nut	PRU 117443
Kirkiaceae	*Kirkia wilmsii* Engl.^.^	Wild pepper	PRU 122536
Malvaceae	*Brachychiton acerifolium* (A. Cunn. ex G.Don) F.Muell	Flame tree	PRU 122529
Malvaceae	*Brachychiton bidwillii* Hook.	Dwarf kurrajong	PRU 122530
Rhamnaceae	*Noltea africana* (L.) Rchb. f.	Soap bush	PRU 122535
Sapindaceae	*Blighia unijugata* Baker	Triangle-tops	PRU 122548

**Table 2:** Minimal inhibitory concentration (MIC in mg/mL) of the leaf extracts from ten plants screened against ten bacterial strains

			MIC: Bacterial strains									
**Plant species**	**Ext**	***Yield (%)***	***E. cl***	***E. c***	***K. p***	***P. m***	***P. a***	***S.*** **T**	***S. m***	***B. c***	***E. f***	***S. a***
*B. a*	M	06.9	0.63	0.63	0.31	0.63	1.25	0.63	1.25	2.50	2.50	2.50
	A	03.5	0.31	0.16	**0.08**	2.50	0.31	0.16	2.50	1.25	0.16	0.31
	E	06.8	0.31	0.16	**0.08**	0.31	0.31	0.16	0.31	1.25	1.25	0.31
	Cw	01.1	1.25	1.25	1.25	2.50	1.25	0.63	2.50	2.50	0.63	1.25
	Hw	13.9	2.50	2.50	0.63	2.50	2.50	0.31	0.63	2.50	2.50	2.50
*B. b*	M	07.0	0.31	0.16	0.63	0.63	0.63	0.31	0.63	2.5	1.25	0.63
	A	03.2	0.16	**0.08**	**0.08**	0.63	0.63	0.31	0.63	1.25	0.16	2.50
	E	10.0	0.31	**0.08**	0.16	1.25	0.63	0.31	0.63	2.5	1.25	1.25
	Cw	01.3	1.25	0.16	2.50	1.25	1.25	2.50	2.50	2.50	2.50	2.50
	Hw	10.0	2.50	2.50	2.50	2.50	2.50	2.50	2.50	2.50	2.50	2.50
*B. u*	M	22.5	0.63	0.31	0.31	0.63	0.16	0.31	0.63	0.16	0.16	0.31
	A	08.5	0.31	**0.08**	**0.08**	0.31	**0.08**	0.63	0.63	**0.08**	**0.08**	0.63
	E	17.2	0.31	0.31	**0.08**	0.31	**0.08**	1.25	0.63	**0.08**	**0.08**	0.63
	Cw	21.5	0.31	0.31	**0.08**	0.31	0.31	0.16	0.63	0.63	1.25	0.31
	Hw	32.2	0.63	0.31	0.31	0.63	0.63	0.63	0.16	0.16	1.25	0.31
*C. m*	M	29.9	0.63	1.25	0.63	1.25	0.16	0.31	0.63	0.16	**0.08**	0.31
	A	10.0	0.63	0.31	0.63	0.31	0.31	0.63	0.63	1.25	0.63	0.31
	E	17.0	1.25	0.63	1.25	0.31	0.31	1.25	0.63	1.25	1.25	0.63
	Cw	18.2	1.25	1.25	1.25	1.25	1.25	1.25	1.25	1.25	2.5	0.63
	Hw	28.1	2.50	1.25	1.25	0.63	1.25	1.25	1.25	1.25	1.25	0.63
*C. b*	M	16.3	1.25	0.31	0.31	0.63	0.63	0.31	0.31	0.63	0.63	1.25
	A	05.1	0.63	0.16	0.16	0.63	0.63	0.16	0.31	0.31	0.16	0.31
	E	06.5	1.25	0.31	0.16	0.63	0.63	0.31	0.63	0.63	0.63	0.63
	Cw	13.2	1.25	0.63	0.31	1.25	0.63	0.63	0.63	0.63	0.63	0.31
	Hw	19.3	1.25	0.31	0.16	0.63	0.63	0.31	0.63	0.63	0.63	0.31
*K. w*	M	24.0	0.16	0.16	**0.08**	0.16	0.63	0.16	0.16	0.31	0.63	**0.04**
	A	06.1	0.63	**0.08**	**0.08**	0.63	0.31	**0.08**	0.16	0.16	0.16	0.16
	E	08.0	1.25	0.31	0.16	1.25	0.63	0.31	0.31	0.63	0.63	1.25
	Cw	10.7	0.63	0.63	0.31	1.25	0.63	0.63	0.63	1.25	0.63	0.63
	Hw	25.6	0.63	0.63	0.63	0.63	0.63	0.63	0.63	0.63	0.63	0.63
*L. a*	M	39.2	**0.04**	**0.04**	**0.08**	**0.08**	0.16	**0.08**	**0.08**	**0.08**	**0.04**	0.16
	A	24.5	**0.04**	**0.04**	**0.08**	**0.04**	0.16	**0.08**	**0.08**	**0.04**	**0.04**	0.16
	E	35.2	**0.08**	**0.08**	**0.08**	**0.04**	0.16	**0.08**	**0.08**	**0.08**	**0.08**	0.16
	Cw	12.0	0.16	0.16	**0.08**	0.16	0.16	0.16	0.16	0.31	0.16	0.16
	Hw	25.8	0.31	0.16	0.31	0.31	0.31	0.31	**0.08**	0.31	0.16	0.31
*N. a*	M	23.8	1.25	0.63	0.63	0.63	0.31	0.31	0.31	0.63	0.31	0.63
	A	06.2	0.16	**0.08**	**0.08**	0.63	0.63	0.31	0.31	0.16	0.31	0.31
	E	09.4	1.25	0.31	0.63	0.63	0.63	0.63	1.25	1.25	0.63	0.63
	Cw	15.9	2.5	1.25	0.63	1.25	1.25	0.63	1.25	0.63	0.63	0.63
	Hw	22.7	1.25	0.63	0.63	1.25	0.63	0.63	0.63	1.25	0.63	0.31
*P. l*	M	22.0	**0.08**	0.16	**0.08**	**0.08**	0.16	0.31	**0.08**	0.16	0.16	**0.04**
	A	10.8	0.16	**0.04**	**0.04**	**0.08**	**0.04**	**0.04**	**0.08**	**0.08**	**0.04**	0.16
	E	14.3	0.16	**0.08**	**0.04**	0.16	**0.08**	**0.08**	0.16	0.16	0.16	0.31
	Cw	15.5	0.63	0.31	0.16	0.31	0.63	0.31	0.31	0.31	0.31	0.63
	Hw	18.0	0.63	0.31	0.16	0.31	0.63	0.16	0.31	0.31	0.31	0.63
*S. l*	M	22.0	1.25	0.31	1.25	0.31	0.31	1.25	0.63	0.31	**0.08**	0.63
	A	04.4	0.16	0.31	0.16	0.31	0.16	**0.08**	0.16	0.16	0.31	0.63
	E	13.8	0.16	0.31	0.16	0.31	0.16	0.16	0.63	0.16	0.31	0.63
	Cw	22.8	0.31	1.25	1.25	1.25	2.5	0.31	2.5	2.5	0.31	0.63
	Hw	23.4	0.31	0.63	0.63	0.63	1.25	1.25	1.25	0.63	0.31	1.25
G			**0.008**	**0.004**	**0.004**	**0.004**	**0.004**	**0.004**	**0.004**	**0.004**	**0.004**	**0.004**
T			**0.063**	**0.004**	**0.004**	**0.008**	**0.016**	**0.004**	**0.008**	**0.002**	**0.001**	**0.002**

*Ext* extract, *ND* not determined, *M* methanol, *E* ethanol, *A* acetone, *Cw* cold water, *Hw* hot water, *B. a* Brachychiton acerifolium, *B. b* Brachychiton bidwillii, *B. u* Blighia unijugata, *C. m* Carissa macrocarpa, *C. b* Combretum bracteosum, *K. w* Kirkia wilmsii, *L. a* Loxostylis alata, *N.a* Noltea africana, *P. l* Protorhus longifolia, *S. l* Searsia leptodictya. *E. cl* Enterobacter cloacae, *E. c* Escherichia coli, *K. p* Klebsiella pneumoniae, *P. m* Proteus mirabilis, *P. a* Pseudomonas aeruginosa, *S. T* Salmonella Typhimurium, *S. m* Stenotrophomonas maltophilia, *B. c* Bacillus cereus, *E. f* Enterococcus faecalis, *S. a* Staphylococcus aureus, *G* Gentamicin, *T* Tetracycline. Values in bold indicate promising activity (MIC < 0.1 mg/mL)

**Fig. 2:**
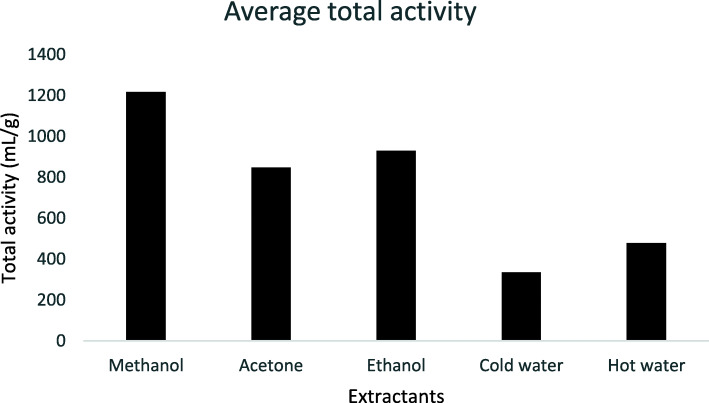
Average total activity for the different extractants against screened bacteria

MIC values ranging from 0.04 to 2.5 mg/mL (Table 3) were observed with further testing of extracts of *L. alata* against the *Salmonella* species: *Salmonella* Typhimurium (ATCC 14028), *S.* Enteritidis (ATCC 13076), *S.* Dublin, *S.* Braenderup *and S.* Typhimurium. The best bacterial inhibition was observed with MIC = 0.04 mg/mL by the methanol crude extract of *L. alata* against *S*. Braenderup (a field isolate from chicken eggs). In ethnopharmacological studies, the quantity extracted from each plant species is as important as the MIC values. Both values were relevant for the determination of the Total Activity (TA). The total activity of the plants ranged from 256.14 to 10,051.28 mL/g (Table 3) for the *Salmonella* serotypes screened in this study. The highest total activity of 10,051.28 mL/g was produced by the methanol extract of *L. alata* against *S*. Braenderup. This implies that 1 g of *L. alata* methanol extract can be diluted in 10,051.28 mL of the solvent used and still inhibit the growth of the bacterium. Considering the average total activity for all extracts, the methanol extract had the best value (Fig. 2) and hence methanol extracts of the selected plant species were used for further assays.

**Table 3:** Extract yield, Minimal Inhibitory Concentration (MIC) values and Total Antibacterial Activity (TAA) of the different leaf extracts of *Loxostylis alata* against selected *Salmonella* spp.

		***Salmonella*** Typhimurium		***Salmonella*** Braenderup		***Salmonella*** Dublin		***Salmonella*** Typhimurium (ATCC 14028)		***Salmonella*** Enteritidis (ATCC 13076)	
Extracts	% yield	MIC (mg/mL)	TAA (mL/g)	MIC (mg/mL)	TAA (mL/g)	MIC (mg/mL)	TAA (mL/g)	MIC (mg/mL)	TAA (mL/g)	MIC (mg/mL)	TAA (mL/g)
Methanol	39.20	0.12 ± 0.06	3350.43	0.04 ± 0.00	10,051.28	0.12 ± 0.06	3350.43	0.16 ± 0.00	2512.82	0.16 ± 0.00	2512.82
Acetone	24.50	0.16 ± 0.00	1570.51	0.16 ± 0.00	1570.51	0.08 ± 0.00	3141.03	0.08 ± 0.00	3141.03	0.16 ± 0.00	1570.51
Ethanol	35.20	0.16 ± 0.00	2256.41	0.47 ± 0.22	751.33	0.12 ± 0.06	3008.55	0.12 ± 0.06	3008.55	0.31 ± 0.00	1128.21
Cold water	12.00	0.31 ± 0.03	384.62	0.47 ± 0.22	256.14	0.16 ± 0.00	769.23	0.16 ± 0.00	769.23	0.31 ± 0.00	384.62
Hot water	25.80	0.31 ± 0.00	826.92	0.47 ± 0.22	550.69	0.23 ± 0.11	1102.56	0.23 ± 0.11	1102.56	0.31 ± 0.00	826.92
Gentamicin	NA	0.008	NA	0.004	NA	0.008	NA	0.008	NA	0.008	NA
Ampicillin	NA	0.004	NA	0.004	NA	0.004	NA	0.004	NA	0.004	NA

*NA* Not applicable

MIC values of the fractions of *L. alata* are presented in Table 4. The MIC values obtained after solvent-solvent fractionation of *L. alata* ranged from 0.04 to 1.25 mg/mL with the ethyl acetate and butanol fractions having the lowest mean MIC values against the *Salmonella* serovars screened (Table 4).

**Table 4:** Minimum inhibitory concentration (MIC) values of fractions of *Loxostylis alata* against *Salmonella* serovars

	MIC (mg/mL)				
Fractions	Salmonella Typhimurium	***Salmonella*** Braenderup	***Salmonella*** Dublin	***Salmonella*** Typhimurium (ATCC 14028)	***Salmonella*** Enteritidis (ATCC 13076)
Hexane	0.16	0.16	0.16	0.16	0.31
Chloroform	0.16	0.16	0.31	0.16	0.31
Butanol	0.08	0.08	0.08	0.08	0.16
Ethyl acetate	0.08	0.04	0.08	0.08	0.16
Water 1	0.08	0.31	0.31	0.16	0.31
Water 2	0.31	0.63	0.63	0.31	1.25
Gentamicin	0.008	0.004	0.008	0.008	0.008
Ampicillin	0.004	0.004	0.004	0.004	0.004

### Antioxidant activity, cytotoxicity and genotoxicity

Almost all the tested extracts had moderate to potent antioxidant activity in both assays (Table 5). Methanol extracts had the highest average total activity compared to other extractants (Fig. 2), and as such were selected for cytotoxicity investigation against Vero monkey kidney cells. Table 6 shows the results for cytotoxicity of crude extracts represented as LC_50_ values, and their respective selectivity index (SI) values where SI = LC_50_/MIC. LC_50_ values > 0.1 mg/mL are considered non-cytotoxic [45]. LC_50_ values of the ten screened extracts ranged from 0.02 to 0.47 mg/mL, while the selectivity index values ranged from 0.03 to 8.05.

**Table 5:** Antioxidant activity of methanol extracts from ten plant species

Plant species	DPPH IC_**50**_ (μg/mL) ± SEM	ABTS IC_**50**_ (μg/mL) ± SEM
*B. acerifolium*	44.96 ± 3.24^d^	33.12 ± 1.68^e^
*B. bidwilli*	10.28 ± 0.41^c^	15.01 ± 0.16^d^
*B. unijugata*	4.14 ± 0.25^a,b,c^	3.07 ± 0.26^a,b^
*C. macrocarpa*	1.13 ± 0.12^a^	3.04 ± 0.02^a,b^
*C. bracteosum*	3.55 ± 0.47^a,b^	6.69 ± 0.16^c^
*K. wilmsii*	4.64 ± 0.58^a,b,c^	2.91 ± 0.11^a,b^
*L. alata*	1.89 ± 0.09^a^	1.35 ± 0.02^a^
*N. africana*	3.06 ± 0.11^a,b^	5.72 ± 0.32^b,c^
*P. longifolia*	5.64 ± 0.00^a,b,c^	2.04 ± 0.03^a^
*S. leptodictya*	7.16 ± 1.12^b,c^	4.21 ± 0.07^a,b,c^
Ascorbic acid (1 mg/ml)	1.59 ± 0.04^a^	1.71 ± 0.32^a^
Trolox (1 mg/ml)	3.04 ± 1.42^a,b^	2.40 ± 0.87^a^

Values with different superscript letters along each column are significantly different at ρ < 0.05. *SEM* Standard error of mean

**Table 6:** LC_50_ and selectivity index values of methanol extracts of selected plant species against Vero monkey kidney cells

Plant species	LC_**50**_ values (mg/mL)	Selectivity index values									
		***E. cl***	***E. c***	***K. p***	***P. m***	***P. a***	***S.*** T	***S. m***	***B. c***	***E. f***	***S. a***
*B. acerifolium*	0.18 ± 0.02	0.29	0.29	0.57	0.29	0.14	0.29	0.14	0.07	0.07	0.07
*B. bidwilli*	0.23 ± 0.04	0.73	**1.45**	0.36	0.36	0.36	0.73	0.36	0.09	0.18	0.36
*B. unijugata*	0.02 ± 0.00	0.03	0.07	0.07	0.03	0.14	0.07	0.03	0.14	0.14	0.07
*C. macrocarpa*	0.47 ± 0.03	0.75	0.38	0.75	0.38	**3.01**	**1.50**	0.75	**3.01**	**6.02**	**1.50**
*C. bracteosum*	0.06 ± 0.00	0.04	0.18	0.18	0.09	0.09	0.18	0.18	0.09	0.09	0.04
*K. wilmsii*	0.03 ± 0.02	0.21	0.21	0.42	0.21	0.05	0.21	0.21	0.11	0.05	0.85
*L. alata*	0.20 ± 0.03	**5.02**	**5.02**	**2.51**	**2.51**	**1.25**	**2.51**	**2.51**	**2.51**	**5.02**	**1.25**
*N. africana*	0.37 ± 0.04	0.30	0.60	0.60	0.60	**1.19**	**1.19**	**1.19**	0.60	**1.19**	0.60
*P. longifolia*	0.31 ± 0.01	**4.02**	**2.01**	**4.02**	**4.02**	**2.01**	**1.01**	**4.02**	**2.01**	**2.01**	**8.05**
*S. leptodictya*	0.24 ± 0.03	0.19	0.77	0.19	0.77	0.77	0.19	0.39	0.77	**3.09**	0.39
Doxorubicin	0.007 ± .003										

*B. acerifolium* Brachychiton acerifolium, *B. bidwilli* Brachychiton bidwillii, *B. unijugata* Blighia unijugata, *C. macrogaba* Carissa macrocarpa, *C. bracteosum* Combretum bracteosum, *K. wilmsii* Kirkia wilmsii, *L. alata* Loxostylis alata, *N. africana* Noltea africana, *P. longifolia* Protorhus longifolia, *S. leptodictya* Searsia leptodictya, *E. cl* Enterobacter cloacae, *E. c* Escherichia coli, *K. p* Klebsiella pneumoniae, *P. m* Proteus mirabilis, *P. a* Pseudomonas aeruginosa, *S. T* Salmonella Typhimurium, *S. m* Stenotrophomonas maltophilia, *B. c* Bacillus cereus, *E. f* Enterococcus faecalis, *S. a* Staphylococcus aureus. Values in bold indicate good activity with promising SI values

As this study primarily sought plant species with good activity against *Salmonella* species, only plants with at least one extract having an MIC value of less than 0.1 mg/mL against *S*. Typhimurium were selected for genotoxicity testing. Hence, *K. wilmsii, L. alata, P. longifolia* and *S. leptodictya* were chosen. The results of the Ames assay for genotoxicity determine if any of the plant extracts had the ability to mutate genes and are presented in Table 7. None of the extracts was genotoxic at the concentrations tested.

**Table 7:** Genotoxicity of selected methanol extracts against *Salmonella* Typhimurium strains (TA98 and TA100) presented as mean ± standard error

Plant species	Dose (mg/mL)	Histidine + revertant colonies	
		TA98	TA100
*Kirkia wilmsii*	5	29.50 ± 0.50	156.00 ± 2.00
	0.5	23.67 ± 2.96	145.67 ± 2.60
	0.05	23.33 ± 0.33	126.00 ± 14.00
*Loxostylis alata*	5	25.67 ± 2.85	201.50 ± 5.50
	0.5	24.67 ± 0.88	188.50 ± 6.50
	0.05	20.33 ± 0.88	145.33 ± 2.33
*Protorhus longifolia*	5	23.33 ± 5.17	163.00 ± 5.00
	0.5	21.00 ± 1.00	135.50 ± 4.50
	0.05	23.50 ± 1.50	142.33 ± 8.69
*Searsia leptodictya*	5	24.67 ± 2.40	172.00 ± 6.00
	0.5	22.33 ± 1.20	157.00 ± 7.00
	0.05	21.67 ± 1.45	109.00 ± 16.00
4-NQO (+ve C)		316.67 ± 7.42	766.00 ± 1.46
10% DMSO (−ve C)		24.67 ± 2.40	158.00 ± 3.04
Water (−ve C)		24.33 ± 2.73	171.00 ± 1.50

*M* methanol extract, *4-NQO* 4-nitroquinoline-1-oxide, *DMSO* Dimethyl sulfoxide, *+ve C* Positive control, *−ve C* Negative control

### Isolation of compounds from *L. alata* with activity against *Salmonella* species

**Fig. 3:**
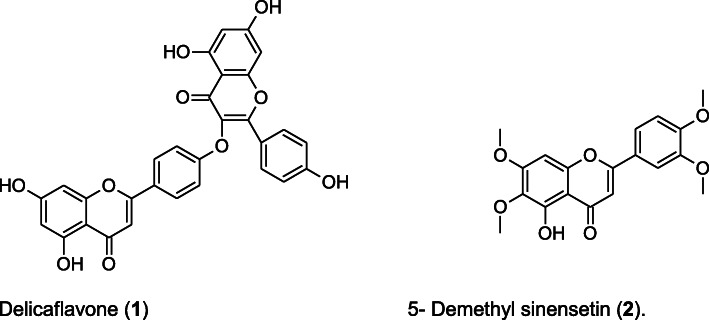
Structure of the compounds delicaflavone (**1**) and ‘5-hydroxy-4’,5′,6,7-tetramethoxyflavone’ (5- demethyl sinensetin) (**2**) isolated from *Loxostylis alata*

Compound **1** (sample 4) was isolated as a whitish amorphous powder and identified by comparing spectroscopic data in available literature as the biflavone compound delicaflavone (3-[4-(5,7-dihydroxy-4-oxo-4H-1-benzopyran-2-yl) phenoxy]-5,7-dihydroxy-2-(4-hydroxyphenyl)-4H-1-benzopyran-4-one) (Fig. 3). Compound **2** (sample 2) was isolated as a whitish amorphous powder and was identified as 5-hydroxy-4′,5′,6,7-tetramethoxyflavone (5-demethyl sinensetin) (**2**) (Fig. 3). Table 8 shows the MIC values obtained for the isolated compounds against the *Salmonella* ATCC strains. Compounds 2 and 3 had the highest MIC values (0.37 mg/mL) followed by compounds 1 and 4 (0.25 mg/mL).

**Table 8:** MIC values of isolated compounds against selected *Salmonella* isolates

Compounds	MIC mg/mL		Average
	***Salmonella*** Typhimurium (ATCC 14028)	***Salmonella*** Enteritidis (ATCC 13076)	
1	0.250	0.250	0.250
2	0.375	0.250	0.312
3	0.375	0.375	0.375
4	0.250	0.250	0.250
Gentamicin	0.008	0.008	0.008
Ampicillin	0.004	0.004	0.004

The remaining samples were not entirely pure and as such they were subjected to GC-MS for identification of their constituents. Table 9 shows the top three chemical constituents with the highest area percentage (> 1%) and over 90% similarity in each sample. The mixtures in samples 3 and 4 were both majorly composed of benzoic acid 3, 4, 5-trihydroxy methyl ester at area percentage values of 39 and 43% respectively. The percentage similarity for both was above 90%, and hence this is a very close match to the reference compound. The GC-MS result for the mixture in sample 5, identified the major constituent as cetene with an area % = 22% (Table 9).

**Table 9:** Percentage composition of the top three chemical constituents of EtOAc fraction of *Loxostylis alata* leaf identified by GCMS

Name	Area %	Molecular Formula	%Similarity	CAS	Sample
*Sample 3:* Benzoic acid, 3,4,5-trihydroxy-methyl ester	**42.669**	C_8_H_8_O_5_	91.9	99–24-1	1
Octadecanoic acid	2.1511	C_18_H_36_O_2_	92.1	57–11-4	1
Decane	1.6976	C_10_H_22_	94.1	124–18-5	1
*Sample 4:* Benzoic acid, 3,4,5-trihydroxy-methyl ester	**39.133**	C_8_H_8_O_5_	91.6	99–24-1	3
Octadecanoic acid	5.7257	C_18_H_36_O_2_	90.6	57–11-4	3
n-Hexadecanoic acid	3.6108	C_16_H_32_O_2_	91.2	57–10-3	3
*Sample 5:* Cetene	**21.732**	C_16_H_32_	92.1	629–73-2	5
Decane	8.7951	C_10_H_22_	90.1	124–18-5	5
1-Penten-3-yne, 2-methyl-	6.0809	C_6_H_8_	93.9	926–55-6	5

The most abundant constituents are highlighted in bold

## Discussion

### Antibacterial activity

A proposed measure suggests that MIC values less than 0.1 mg/mL are considered as good antimicrobial activity; MIC of 0.1 to 0.5 mg/mL is moderate antimicrobial activity while MIC of 0.5 to 1 mg/mL is weak antimicrobial activity and MIC of greater than 1 mg/mL is considered inactive [46]. The extracts of *L. alata* and *P. longifolia* had promising results with good (MIC < 0.1 mg/mL) antibacterial activity in at least three out of the five extracts tested and growth inhibition of at least eight out of the ten (80%) bacteria screened. The results in this study confirm the good antibacterial activity of *L. alata* as reported in a previous study, where extracts of this plant had MIC values as low as 0.08 mg/ml against some bacteria including *E. coli* [47]. The significant activity (MIC value between 0.04 and 0.08 mg/mL) against *E. coli* obtained for the acetone extracts of *B. bidwillii*, *B. unijugata*, *K. wilmsii*, *L. alata*, *N. africana* and *P. longifolia* is noteworthy. It should be kept in mind that antibacterial activity in vivo may also be attributed to specific enzyme activation, immunomodulation or immune stimulation [44, 48] rather than direct bacterial growth inhibition.

Further testing of extracts of *L. alata* against *Salmonella* species identified very good activity against *Salmonella* Braenderup of the methanol crude extract of *L. alata*. This extract also had the best total activity against *S*. Braenderup. In general, the total activity of the methanol extracts was the best, hence the choice of methanol as extractant for the bulk extraction of *L. alata*. The ethyl acetate fraction produced using solvent-solvent fractionation had strong activity against the *Salmonella* serovars, and was therefore selected for isolation of active compounds.

### Antioxidant activity, cytotoxicity and genotoxicity

The ABTS and DPPH assays provide a rapid and efficient in vitro evaluation of antioxidant capacity of plant phytochemicals [49]. Several plant species including *L. alata* and *P. longifolia* had promising antioxidant activity. A plant extract-based preparation with various functions including antioxidant (to prevent chemical spoilage of food) and antimicrobial (active against both pathogenic and food spoilage organisms) could be useful in the food and health industries, potentially enabling a reduction in the amount of synthetic additives used in foods [50].

A varying range of cytotoxicity of the methanol extracts was observed in this study, and the best selectivity index value of 8.05 was demonstrated by *P. longifolia*. A high selectivity index value suggests a good safety margin between the concentration of test sample able to inhibit the bacteria, and the concentration toxic to mammalian cells [44]. None of the extracts with good antibacterial activity were genotoxic in this study.

### Isolation of compounds from *L. alata* with activity against *Salmonella* species

Two compounds were isolated and identified using mass spectrometry and NMR as delicaflavone and 5-demethyl sinensetin. The major components in the other two samples isolated were analysed using GC-MS, with benzoic acid 3, 4, 5-trihydroxy methyl ester being the primary compound identified. A search of the compound “benzoic acid, 3,4,5-trihydroxy methyl ester” on the National Center for Biotechnology Information (PubChem) Compound Database, revealed that benzoic acid, 3,4,5-trihydroxy methyl ester, is also known as methyl gallate. Sample 5 comprised mostly cetene, or 1-hexadecene, a chemical constituent of surface active agents, laundry and dish washing products [51]. Hence compound 5 was majorly a contaminant.

*Loxostylis alata* of the family Anacardiaceae [52] is used traditionally in South Africa for therapeutic purposes. The bark and leaves of this plant are used during childbirth [52] and, interestingly, to stimulate the immune system [53]. Several compounds such as 3-(8Z-pentadecenyl) phenol (ginkgol) and 6-(8Zpentadecenyl) salicylic acid (ginkgolic acid) from the bark [54], lupeol and β-sitosterol have been isolated from *L. alata* leaves [55]. Overall, the antibacterial activity of the compounds was lower than that of the ethyl acetate fraction. The logical explanation would be that the observed activity for the crude extract or fractions occurs as a result of the synergy of the numerous constituents [44].

Biflavonoids are rare, polyphenolic molecules comprised of two identical or non-identical flavonoid units conjoined in a symmetrical or asymmetrical manner through alkyl or alkoxy-based links of varying length [56, 57]. Biflavones have been reported in various studies to exhibit strong bioactivities, such as vasorelaxation, antifungal, antiviral, anti-inflammatory, anticancer bioactivities, inhibition of tumour metastasis [57–60], and they also exhibit good antioxidant properties [61, 62]. When present in plants, these biflavonoids are considered to be the main active ingredients accounting for the treatment effect of those herbal medicines [57]. Delicaflavone is a rarely occurring biflavonoid [63], and has previously been isolated from some *Selaginella* species [57, 64, 65]. A previous study has also reported that delicaflavone exhibited favourable anticancer properties with anti-lung cancer effects in vitro and in vivo by inducing autophagic cell death via the Akt/mTOR/p70S6K signalling pathway [63]. Delicaflavone did not have observable side effects in a xenograft mouse model [63]. These properties, therefore, enhance the potential of the compound, or its derivatives, as a therapeutic agent.

The compound 5-demethyl sinensetin belongs to the group of flavones referred to as ‘polymethoxyflavones’ (PMFs) which are almost exclusively found in the citrus genus [66]. The polymethoxyflavones have been extensively studied for their anticancer potential against a number of cancer cell lines [66–69]. The anti-inflammatory ability of PMFs has also been demonstrated in a previous study [67, 68]. Results obtained from screening PMFs for cell proliferation and apoptosis induction in HL-60 cancer cell lines, showed “moderate to strong activities against the proliferation and induced apoptosis of HL-60 cell lines” [66]. A combination of inhibition effect and strong apoptosis property was obtained for 5-demethylated PMFs, which is a desirable anti-cancer property [68]. A study [69], demonstrated the ability of 5-demethyl sinensetin (as well as other polymethoxyflavones) to induce differentiation of normal human epidermal keratinocytes, an essential stage in the epidermal role as a barrier to water loss and microbial invasion [69]. Hence, 5-demethyl sinensetin is an appealing candidate to explore for anti-cancer activities.

Methyl gallate is a gallotannin (phenolic compound) which has been reported to be widely distributed in nature [70, 71]. Polyphenols and flavonoids have been associated with modulating immune response [72], and hence there has been growing interest in the study of these classes of compounds. Methyl gallate has been isolated from plants used traditionally to treat gastrointestinal disorders, for example the stem bark of *Entada abyssinica* [73] and leaves of *Pimenta racemosa* [74]. Previous studies have demonstrated its bioactivities such as antioxidant [75–77], anti-inflammatory [74, 78–80], antibacterial [70, 73, 81, 82] and anti-tumor [83, 84]. Interestingly, some of the antibacterial studies on methyl gallate were able to demonstrate the mechanism of action against the pathogens. In a study on the inhibitory effect of methyl gallate on the plant-pathogenic bacterium *Ralstonia solanacearum* [70], scanning electron microscopy revealed that methyl gallate exerted damage to the cell wall structure of the pathogen, causing morphological changes and plasmoptysis [70]. In the same study, Fan et al. (2014) also reported that methyl gallate exhibited inhibition of protein synthesis and succinate dehydrogenase (SDH) activity in *Ralstonia solanacearum*. Another study worth noting investigated the role of methyl gallate in adhesion, invasion, and intracellular survival of *Salmonella* Typhimurium [82]. In the study by Birhanu et al. (2018), the researchers reported the ability of methyl gallate alone or in combination with sub MIC of marbofloxacin to inhibit the adhesion, invasion, motility, and intracellular survival of *S*. Typhimurium. This potential was reportedly due to the ability of methyl gallate to down-regulate quorum sensing and virulence genes in *S*. Typhimurium [82].

## Conclusion

The increase in foodborne infection with its associated economic impact cannot be over-emphasized, hence the continuous search for alternative, safe and affordable therapeutics. This study investigated the in vitro antibacterial activity of selected plant species against a range of bacteria implicated in causing diarrhoea. The plant extracts tested had varying levels of activity against bacteria. This is the first report of antibacterial potential of *B. acerifolium* and *B. bidwillii*. Good antibacterial and antioxidant activity exhibited by the extracts of *L. alata* and *P. longifolia* provides support for the traditional use of these plant species as immune boosters, motivating further studies in this regard. However, cytotoxicity of extracts from *B. unijugata, C. bracteosum* and *K. wilmsii* suggests that these species should be used with caution in traditional medicine. *Loxostylis alata* exhibited good antibacterial activity against all bacteria tested, and had the highest selectivity index against *Salmonella* Typhimurium, providing motivation for further investigation to determine if the plant could be a source of an antibacterial drug lead against *Salmonella*. Results obtained support previous reports of the antibacterial potential of extracts of *L. alata*. However, its anti-*Salmonella* potential is reported for the first time in this study. This is also the first report of a biflavone (delicaflavone), polymethoxyflavone (5-demethyl sinensetin) and gallotannin (methyl gallate) isolated from *L. alata*. The isolated compounds did not possess exceptional anti-*Salmonella* potential individually, so the activity of the total extract may be attributed to synergistic properties of the plant secondary metabolites. However, these compounds may be structurally modified to enhance their activity. Combined with its good antioxidant activity and low in vitro toxicity, the antimicrobial efficacy of *L. alata* warrants further studies on its potential in protecting food against contamination and spoilage, as well as against diseases caused by foodborne pathogens.

## Data Availability

Not applicable.

## Abbreviations

ABTS: 2, 2–azino-bis- (3-ethylbenzothiazoline-6-sulfonic acid)
ATCC: American Type Culture Collection
CC: Column chromatography
DMSO: Dimethyl sulfoxide
DPPH: 2, 2′-diphenyl-1-picrylhydrazyl
EDTA: Ethylenediamine tetraacetic acid
GC-MS: Gas chromatography-mass spectrometry
EtOAc: Ethyl acetate
INT: Iodonitrotetrazolium chloride
iNTS: invasive non-typhoidal *Salmonella*
MEM: Minimal Essential Medium
MeOH: Methanol
MH: Müller-Hinton
MIC: Minimum inhibitory concentration
MTT: 3-(4, 5-dimethylthiazolyl-2)-2.5-diphenyltetrazolium bromide
NMR: Nuclear magnetic resonance
4-NQO: 4-nitroquinoline-1-oxide
PMF: Polymethoxyflavone
SI: Selectivity index
TAA: Total Antibacterial Activity
TLC: Thin layer chromatography
UPLC: Ultra Performance Liquid Chromatography
XLD: Xylose lysine deoxycholate
